# Supplementary material for: Structural analysis of human CEACAM1 oligomerization
Source: Commun Biol. 2022 Sep 30;5:1042. doi: 10.1038/s42003-022-03996-4 (PMC9525683; doi:10.1038/s42003-022-03996-4)
Supplement: Supplementary file 2 — Supplementary Information [file 42003_2022_3996_MOESM2_ESM.docx]

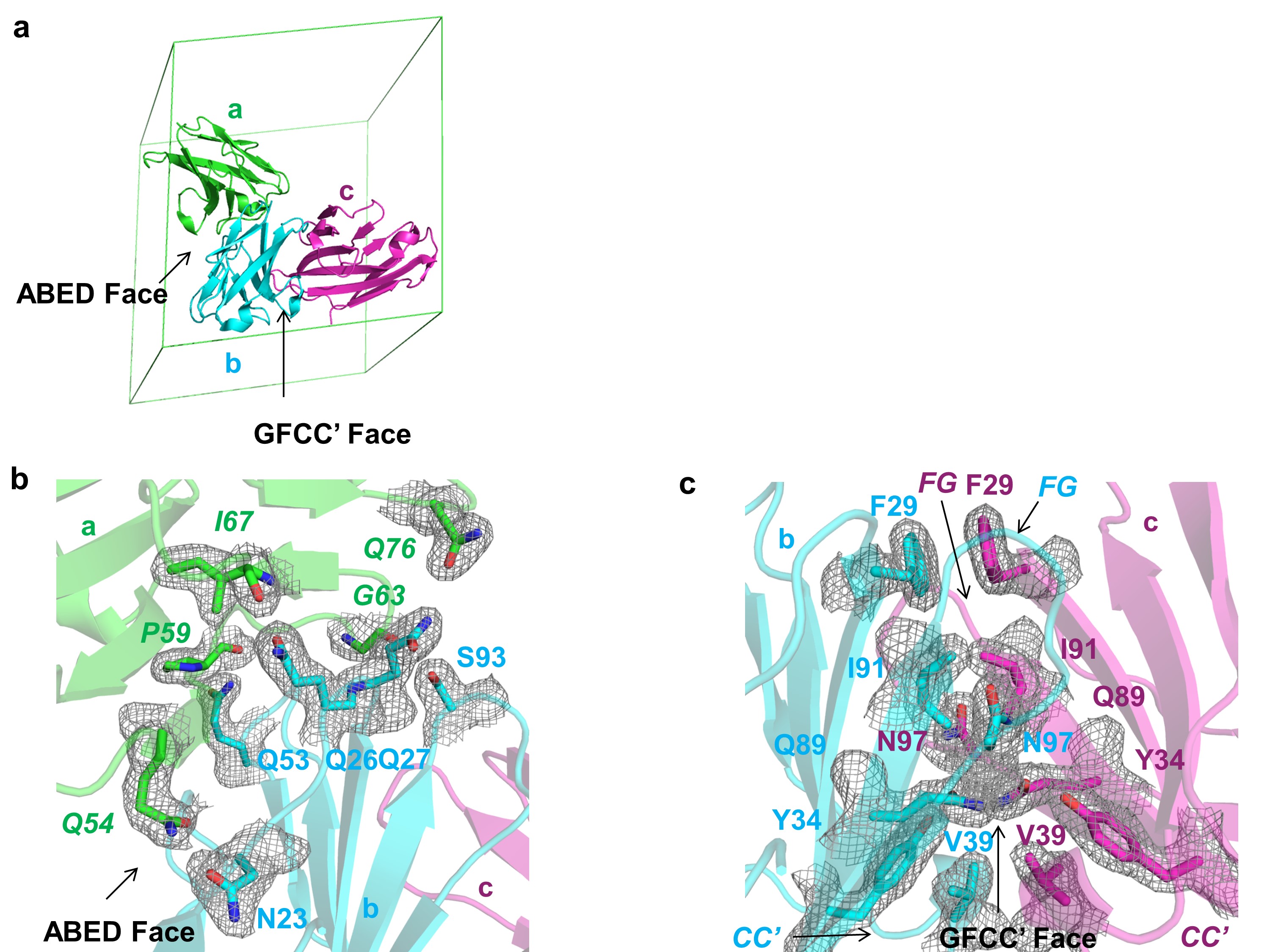


**Supplementary Fig. 1. hCEACAM1 oligomer crystal structure (PDB code 7RPP, resolution 2.20 Å) with simultaneous interactions of GFCC’ and ABED faces.** **a** Ribbon diagram of the three hCEACAM1 molecules (a, b, c) as observed in the crystal asymmetric unit making GFCC’ and ABED face interactions (highlighted by arrows). Crystal asymmetric unit is shown and hCEACAM1 molecules a, b and c are colored in green, cyan and magenta, respectively. **b** Snapshot of the ABED face interactions with electron density as observed between molecules a (green) and molecule b (cyan) residues in the crystal structure, wherein molecule *a* residues *Q54, P59, G63, I67, Q76* (green) make six hydrogen-bond interactions with molecule b residues N23, Q26, Q27, Q53, S93 (cyan). Residues are shown by sticks with corresponding electron density (2Fo–Fc map at 1.0 σ level). The carbon atoms in green (molecule a) or cyan (molecule b), carbonyl oxygen in red and nitrogen in blue, are colored respectively. **c** Snapshot of the GFCC’ face interactions with electron density as observed between molecules b (cyan) and molecule c (magenta) residues in the crystal structure, wherein molecule *b* residues F29, Y34, V39, Q89, I91, N97 (cyan) make various hydrogen-bond and hydrophobic interactions with molecule a residues F29, Y34, V39, Q89, I91, and N97 (magenta). Residues are shown by sticks with corresponding electron density (2Fo–Fc map at 1.0 σ level). The carbon atoms in cyan (molecule b) or magenta (molecule a), carbonyl oxygen in red and nitrogen in blue, are colored respectively. *CC’* and *FG* loops are labeled accordingly.


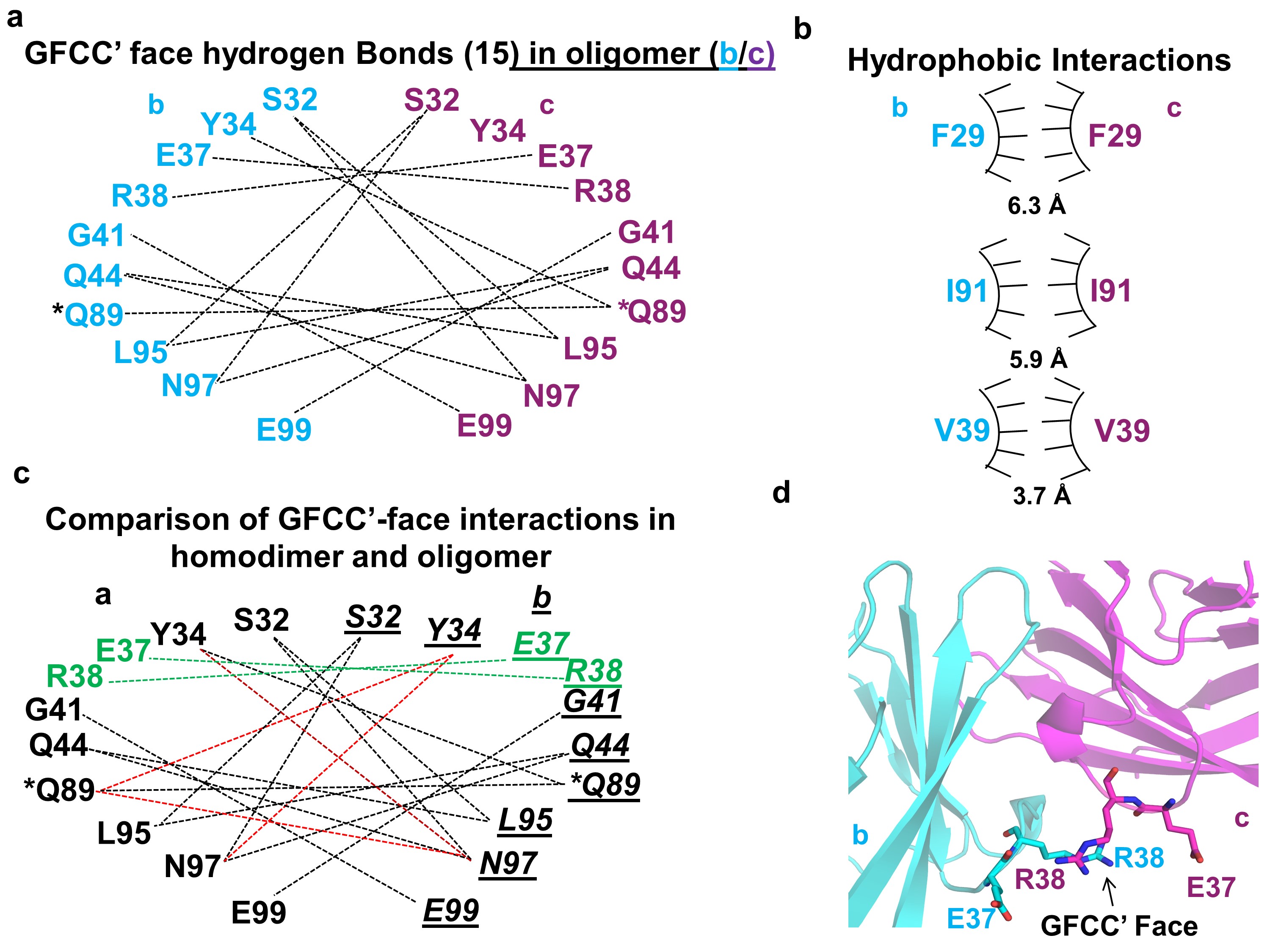


**Supplementary Fig. 2. GFCC’ face-mediated hydrogen-bond and hydrophobic interactions as observed in hCEACAM1 oligomer crystal structure (PDB code 7RPP) and comparison with WT homodimer (PDB code 4QXW). a** Fifteen (15) hydrogen-bond interactions through GFCC’ face residues as observed in the crystal structure between molecule b (cyan) and molecule c (magenta) residues. The hydrogen-bond interactions (15) across the GFCC’ face by labelled residues described are shown by dashed lines. The asterisk (*) indicates two hydrogen-bonds (shown by single dashed line) mediated by Q89 residues of molecules b and c with each other via OE1 and NE2 atoms. Similar hydrogen-bond interactions were also observed in previously reported crystal structure of hCEACAM1 IgV domain (PDB code 4QXW) with exceptions of E37-R38 and N97A symmetrical interactions observed here. **b** Hydrophobic interactions at GFCC’ face through F29, I91 and V39 residues are shown by arc/point representations. The molecule (a) and (b) residues are labeled with hydrophobic interactions as measured by distance between β carbons of labeled residues of molecules b and c. Similar hydrophobic interactions were also observed in previously reported crystal structure of hCEACAM1 Ig-V domain (PDB code 4QXW). **c** Comparison of hydrogen-bond interactions through GFCC’ faces of previously reported dimer (PDB code 4QXW) and oligomer (PDB code 7RPP). Seventeen (17) hydrogen-bond interactions through GFCC’ face residues are observed in the WT homodimer crystal structure (PDB code 4QXW) between molecule a and molecule b (underlined) residues. The 17 hydrogen-bond interactions across the GFCC’ face by labelled residues described are shown by black and red dashed lines. The interactions shown by dashed red lines were not observed in hCEACAM1 oligomeric crystal structure. The interactions shown by green dashed lines between E37-R38 residues were observed only in oligomeric crystal structure, and not observed in homodimer crystal structure (PDB code 4QXW). The asterisk (*) indicates two hydrogen-bonds (shown by single dashed line) mediated by Q89 residues of molecules a and b with each other via OE1 and NE2 atoms. **D** Ribbon diagram of the hCEACAM1 crystal structure reported here with GFCC’ face, where additional interactions between E37 and R38 residues of molecules b (cyan) and c (magenta) were observed compared to previously reported crystal structure of hCEACAM1 IgV domain (PDB code 4QXW). E37 and R38 residues are shown by sticks.


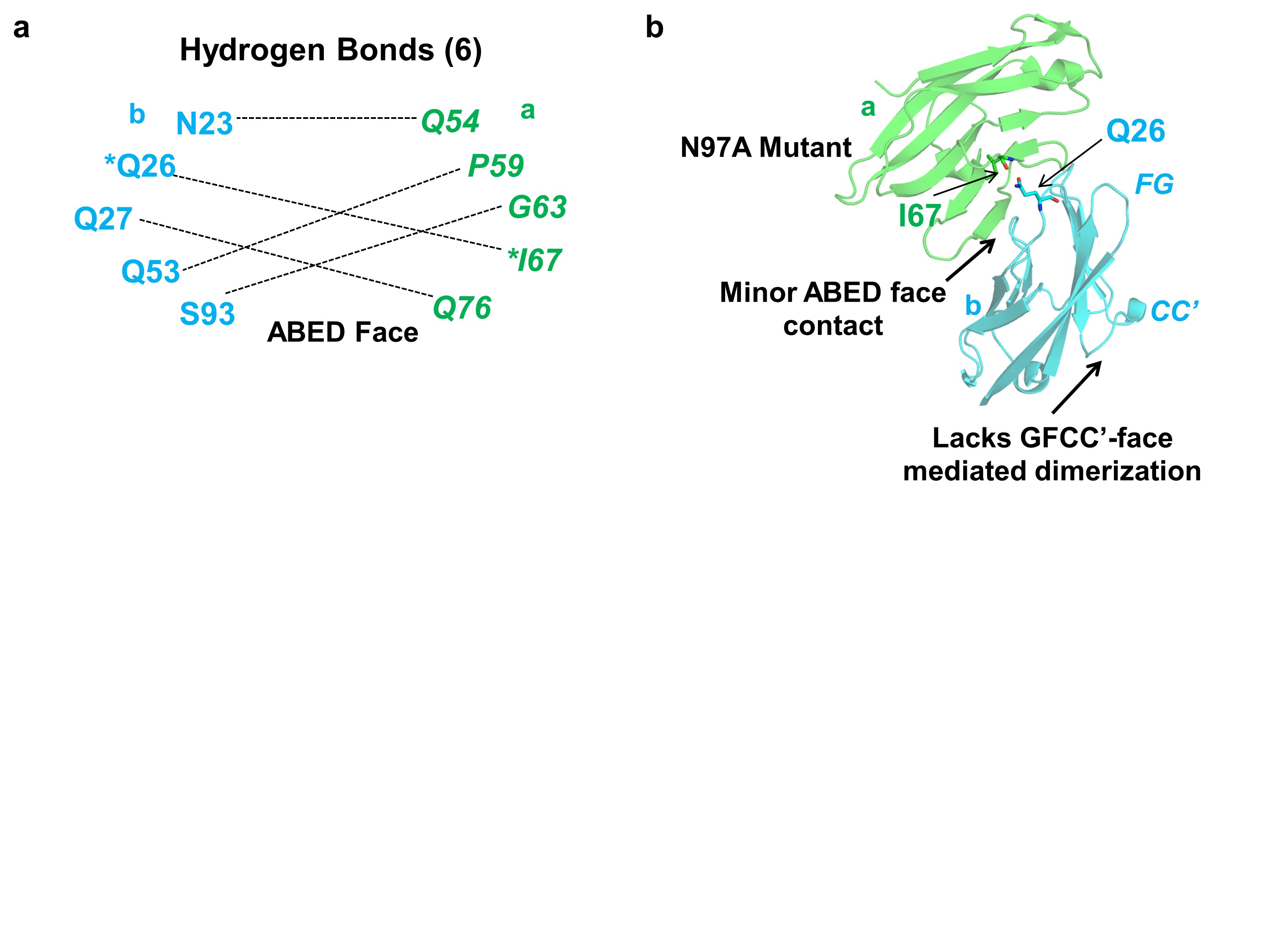


**Supplementary Fig. 3.** **ABED face-mediated hydrogen-bond interactions as observed in hCEACAM1 oligomer crystal structure (PDB code 7RPP). a** Six hydrogen-bond interactions (shown by dashed lines) observed at the ABED face by labeled residues. The residues of molecules (b) and (a) are shown in bold cyan and green italics, respectively. The asterisk (*) indicates two hydrogen-bonds mediated by molecule b residue Q26 and molecule c residue I67. **b** Crystal structure of N97A monomeric mutant (PDB code 6XO1), which lacks GFCC-face mediated dimerization and showed very minor contact at the ABED interface that was restricted to the Q26-I67 residues (stick representation) between two hCEACAM1 molecules (a in green and b in cyan). CC’ and FG loops in molecule b are labeled in italics.

.


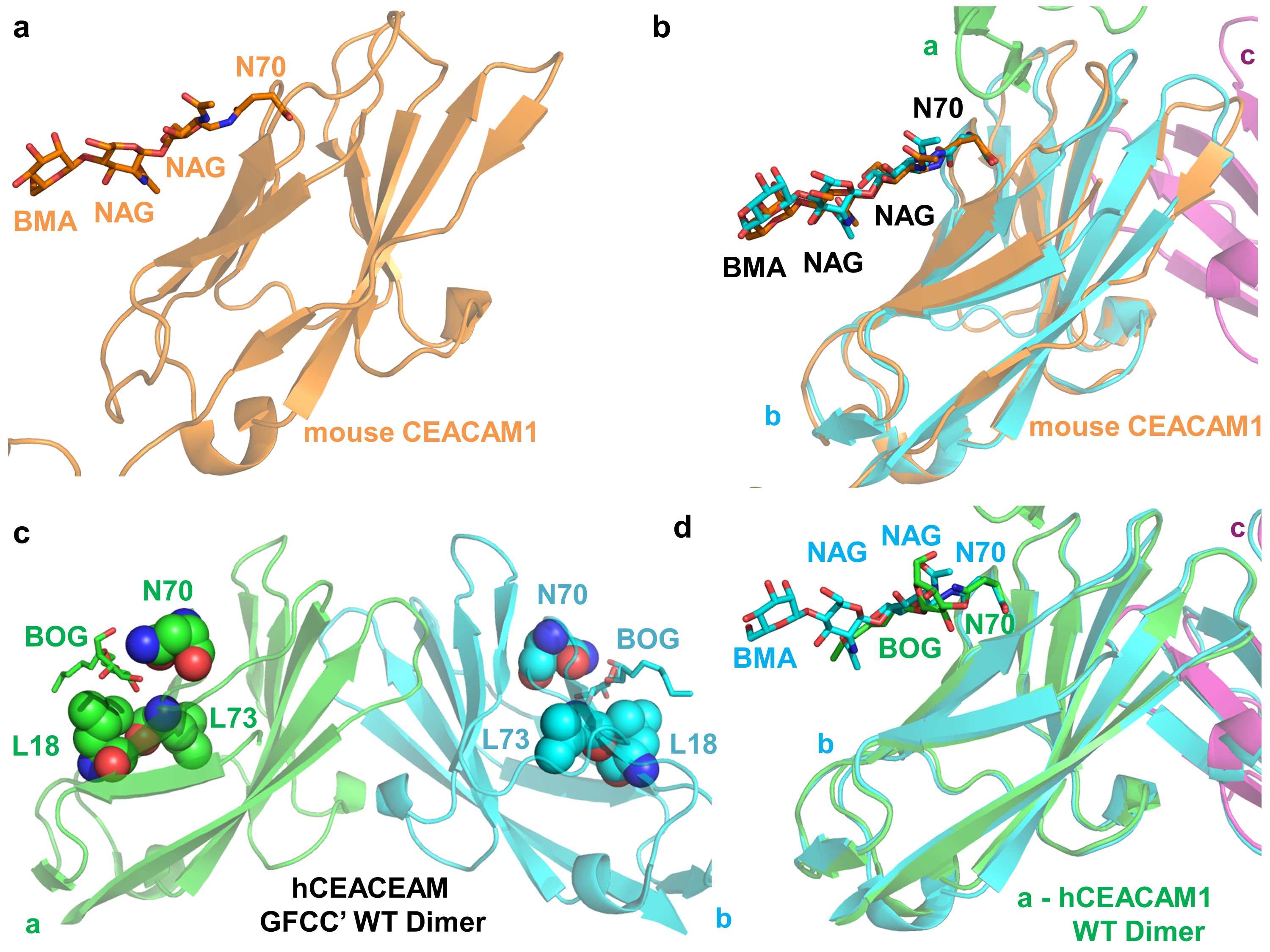


**Supplementary Fig. 4. Structural methodology for glycosylated model of the hCEACAM1. a** Ribbon diagram of the mouse CEACAM1 crystal structure (PDB code 1L6Z) wherein conserved N70 glycosylation site residue was bound to two molecules of N-acetylglucosamine (NAG) and a molecule of β-d-Mannose (BMA) shown by sticks. **b** Modeling of the two NAG and one BMA molecules (shown by cyan sticks) onto the N70 residues of molecule b (cyan) in the crystal structure reported here based on the superimposition of the mouse CEACAM1 crystal structure with N70 residue N-linked NAG-NAG-BMA sugar molecules (shown by orange ribbon diagram and sticks). **c** Ribbon diagram of the human CEACAM1 crystal structure (PDB code 4QXW) wherein octyl beta-D-glucopyranoside (BOG, shown by green and blue sticks for molecules a and b) was observed to bind near L18, N70 and L73 residues (shown by spheres for molecules a and b). **d** Superimposition of the human CEACAM1:BOG bound crystal structure molecule a (green, PDB code 4QXW) onto the N70 glycosylated model of molecule b (cyan) shown above, wherein BOG superimposed very well on the N70 linked modeled sugar molecules. Based on this methodology and final N70-linked model sugars, other N70, N77 and N81 residues of molecules a, b and c were modeled with two molecules of NAG and one molecule of BMA as well.


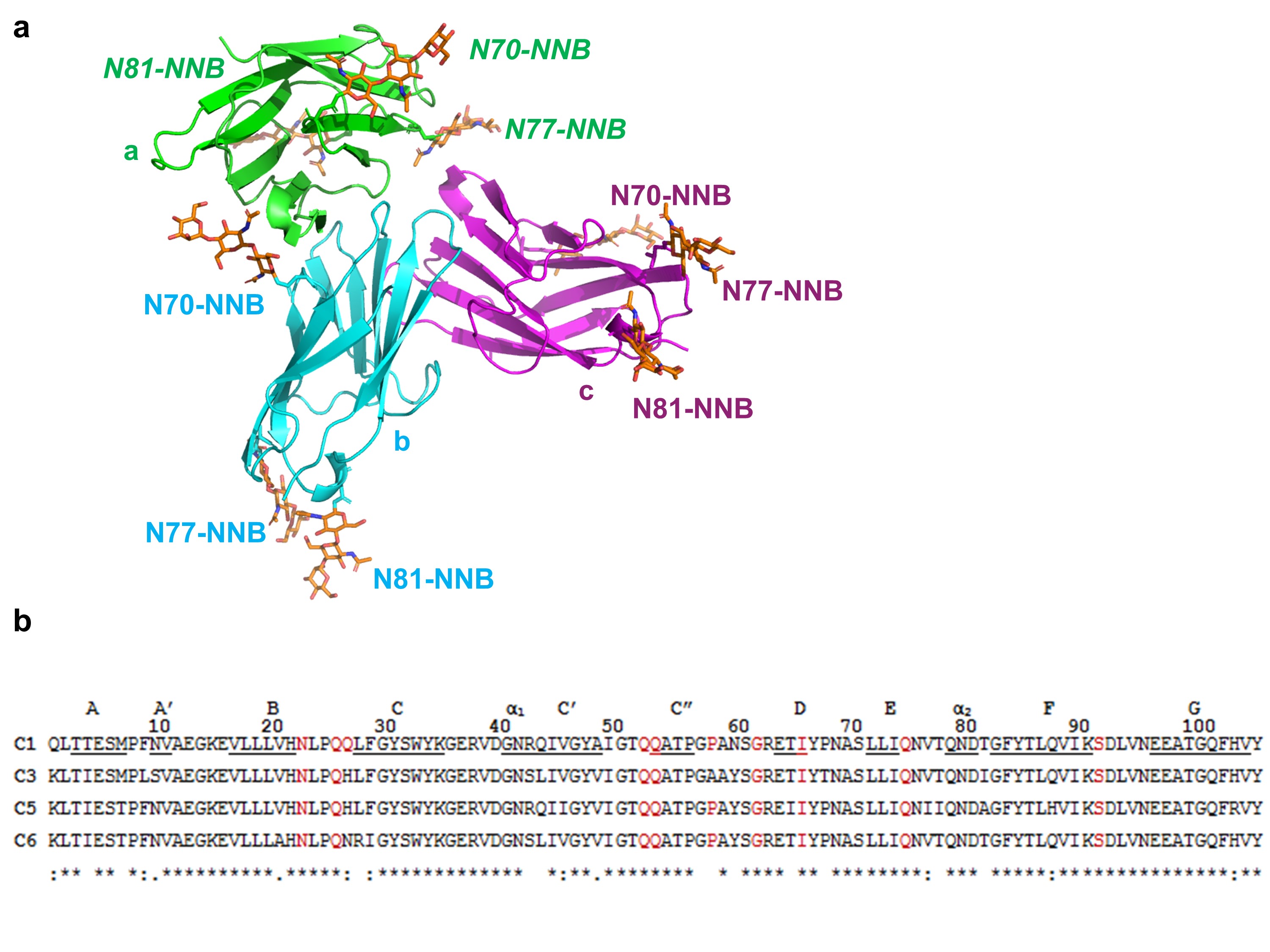


**Supplementary Fig. 5. Glycosylated model of hCEACAM1 and IgV domain sequence homology among human CEACAM family members. a** Modeled N-linked sugars N-acetylglucosamine (NAG) and β-d-Mannose (BMA) onto the N70, N77, and N81 residues of human CEACAM1 molecules using the CHARMM glycan server. **b** The IgV domain sequence alignments of hCEACAM1 (C1), CEACAM3 (C3), CEACAM5 (C5), and CEACAM6 (C6). The residues which participated in ABED face interactions are mostly conserved, colored red and includes four conserved glutamine residues (Q26, Q53, Q54 and Q76). The nine β strands and two α helices are labeled and underlined. Asterisk (*) indicates identical residue conserved across all family members. Period (.) indicates conservation of weakly similar residues, and a colon (:) indicates conservation of strongly similar residues.


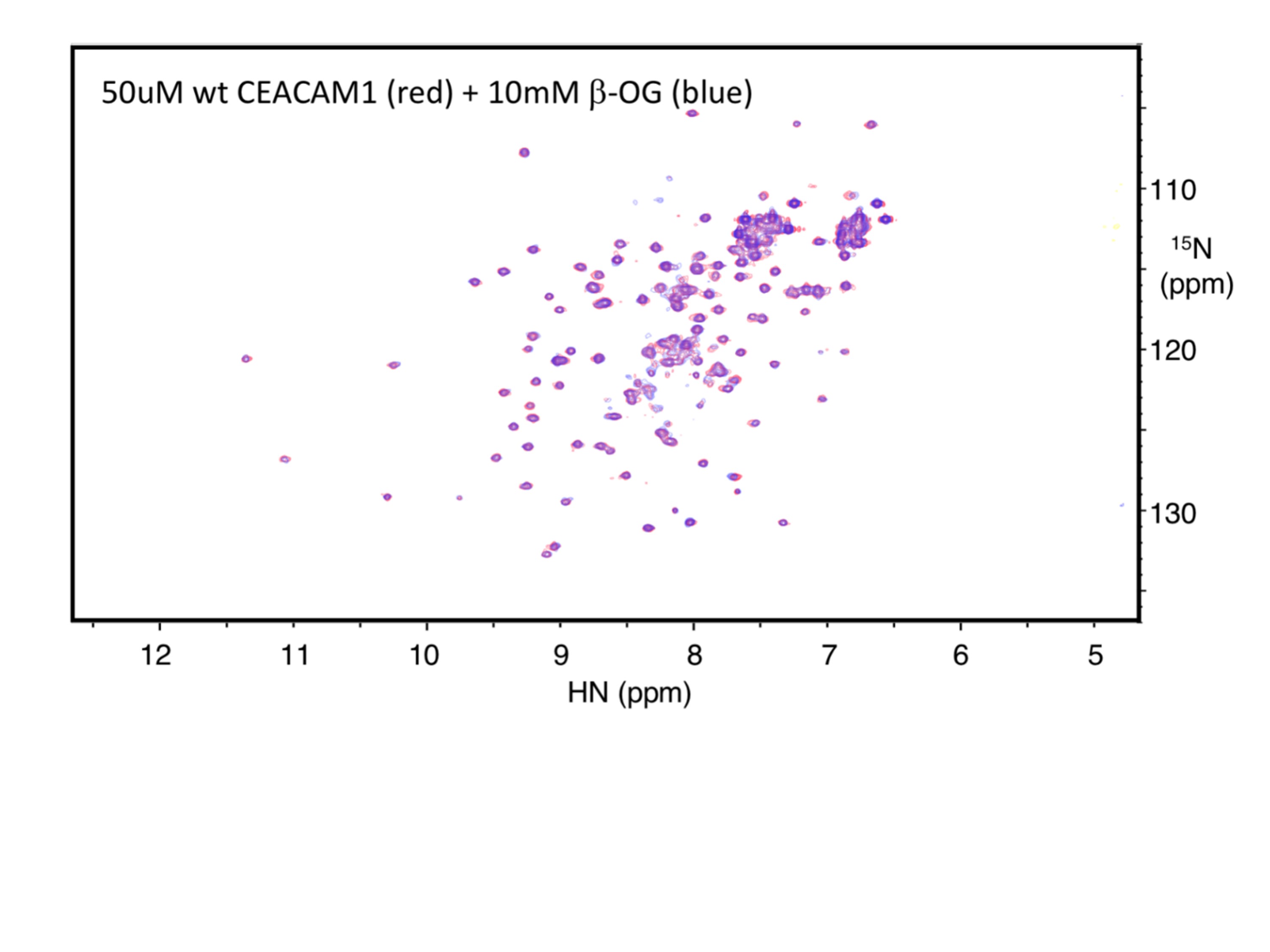


**Supplementary Fig. 6**. **^15^N-HSQC spectra of wild type hCEACAM1 IgV domain (50 µM) binding with 10 mM octyl beta-D-glucopyranoside (BOG).** Overlaid ^15^N-HSQC spectra of hCEACAM1 WT alone (blue) and with BOG (red) reveal largely similar structures.


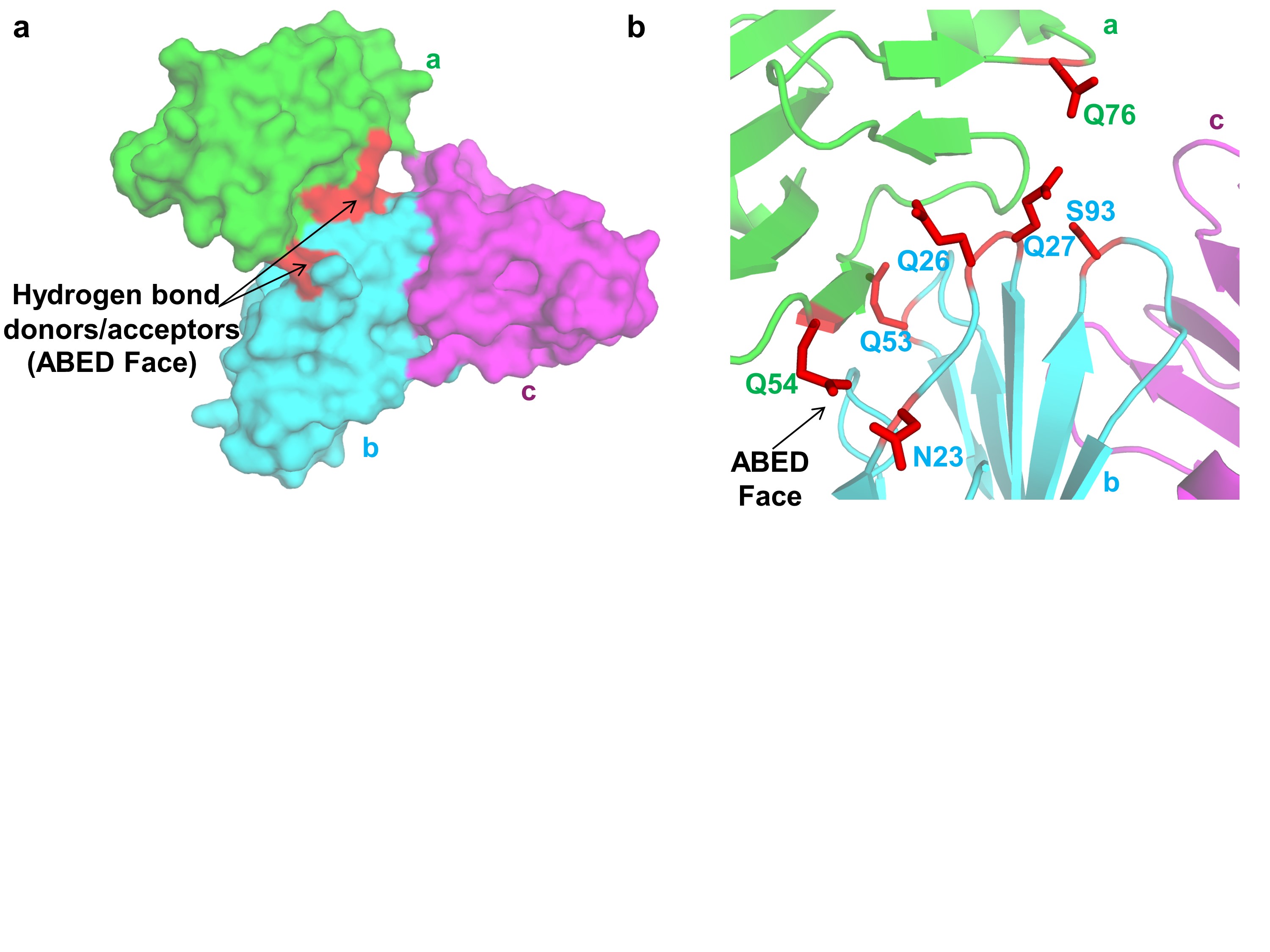


**Supplementary Fig. 7**. **Human CEACAM1 higher order oligomerization is supported by flexible ABED interactions. a** Surface diagram of the hCEACAM1 crystal structure reported here with dual adhesion and interacting GFCC’ and ABED interfaces. The residues N23, Q26, Q27 Q53, Q54, Q76, S93 which participated in the ABED face interactions and have dual hydrogen-bond donor and hydrogen-bond acceptor properties are shown by red colored interface. **b** Ribbon diagram of the hCEACAM1 crystal structure reported here. The residues N23, Q26, Q27 Q53, Q54, Q76, S93 which participate in the ABED face interactions are shown by red colored sticks.


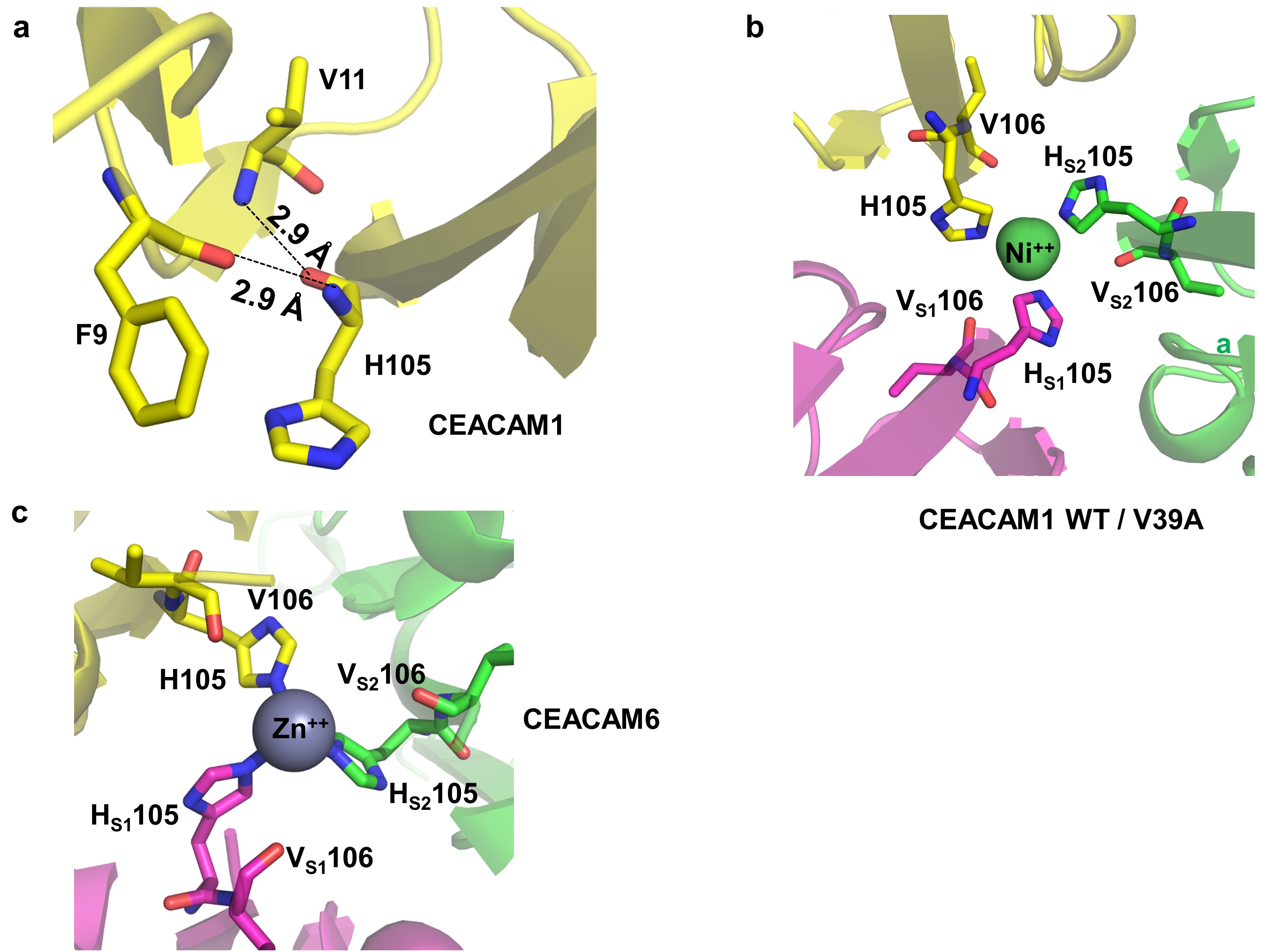


**Supplementary Fig. 8**. **Human CEACAM1 bridging with metal ions (Zn^++^ and Ni^++^). a** Stick representation of FG loop and G strand hCEACAM1 residues H105, F9 and V11 making two ~2.9 Å hydrogen-bond interactions (shown by dashed lines). The residues are labeled, whereas the carbon atoms in yellow, carbonyl oxygen in red and nitrogen in blue, are colored, respectively. **b** Binding of Ni^++^ with hCEACAM1 H105 and V106 residues as observed in the hCEACAM1 WT (PDB code 2GK2) and V39A mutant (PDB code 6XNW), wherein hexadentate interactions by His105 and Val106 residues of three symmetry-related hCEACAM1 molecules with Ni^++^ was observed. Residues are shown by sticks and Ni^++^ is shown by green sphere. H105 and V106 residues of symmetry molecule 1 (in cyan) are labeled as H_S1_105 and V_S1_106, and H105 and V106 residues of symmetry molecule 2 (in green) are labeled as H_S2_105 and V_S2_106. **c** Similar hexadentate interactions mediated by His105 and Val106 residues with Zn^++^ as observed in the human CEACAM6 crystal structure (PDB code 4Y8A). Zn^++^ is shown by blue sphere. H105 and V106 residues are shown by sticks and labeled as above.


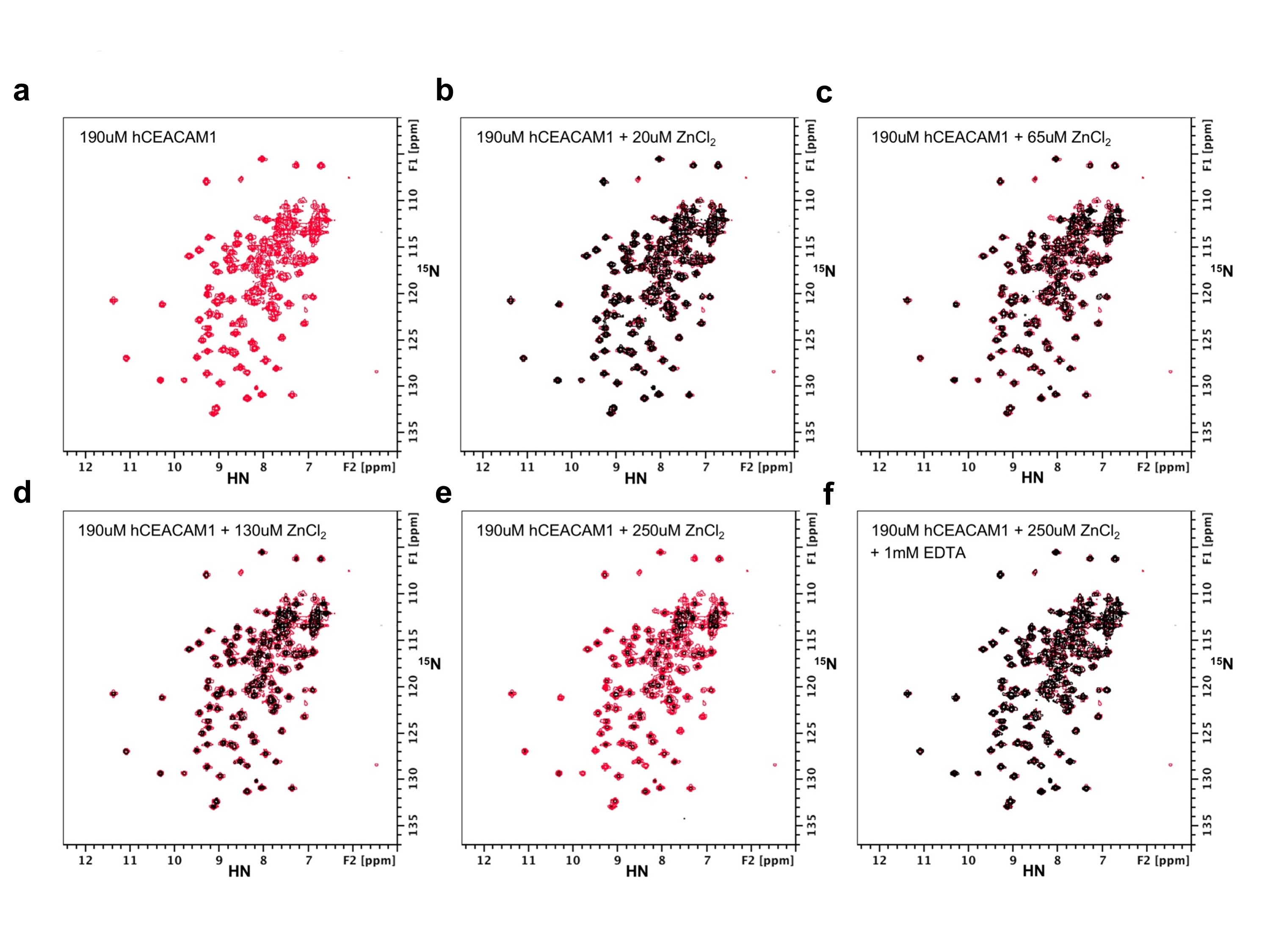


**Supplementary Fig. 9**. **^15^N-HSQC spectra of hCEACAM1 WT IgV with zinc chloride (ZnCl_2_) and effect of EDTA.** ^15^N-HSQC spectra of 190 μM ^15^N-labeled WT hCEACAM1 IgV domain alone (panel a, peaks in red), and after separately titrating 20 μM, 65 μM, 130 μM, and 250 μM ZnCl_2_ (panels b-e, weakened peaks in black) and finally with both 250 μM ZnCl_2_, and 1 mM EDTA (panel f, recovered peaks in black).


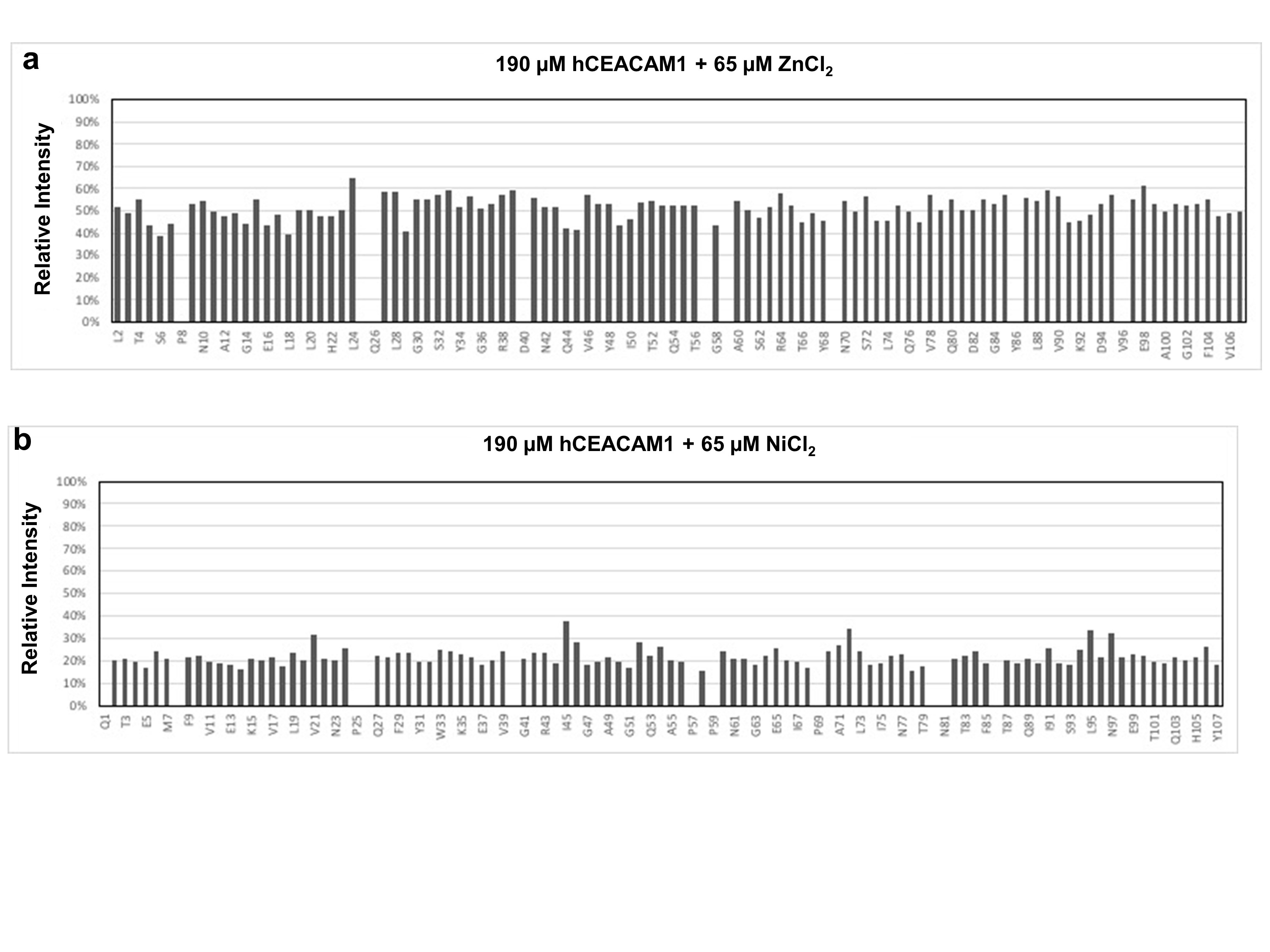


**Supplementary Fig. 10**. **^15^N-HSQC spectra of hCEACAM1 WT IgV with zinc chloride (ZnCl_2_) or nickel chloride (NiCl_2_) showing NMR signal loss caused by addition of nickel or zinc is nearly uniform of all residues. a** Relative peak intensity of CEACAM1 residues with/without 65 µM ZnCl_2_. **b** Relative peak intensity of CEACAM1 residues with/without 65 µM NICl_2_.


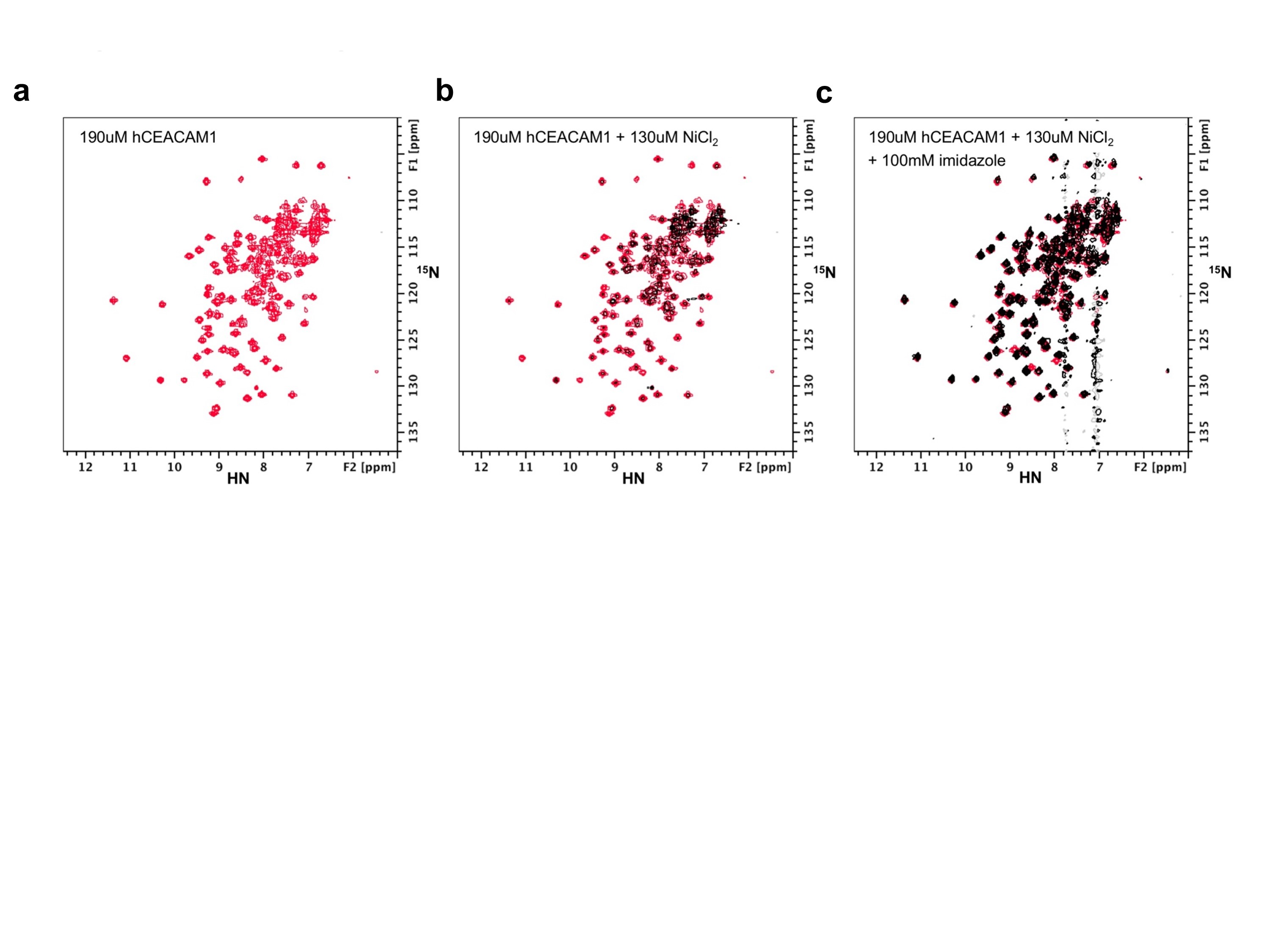


**Supplementary Fig. 11**. **^15^N-HSQC spectra of hCEACAM1 WT IgV with nickel chloride (NiCl_2_) and effect of Imidazole.** ^15^N-HSQC spectra of 190 μM ^15^N-labeled WT hCEACAM1 IgV domain alone (panel a, peaks in red), and in the presence of 130 μM NiCl_2_ (panel b, weakened peaks in black), and both 130 μM NiCl_2_ and 100 mM imidazole (panel c, recovered peaks in black). The weakened backbone amide peaks caused by nickel bridging are recovered after addition of free imidazole to compete with histidine residues and support observation of the crystal structures.


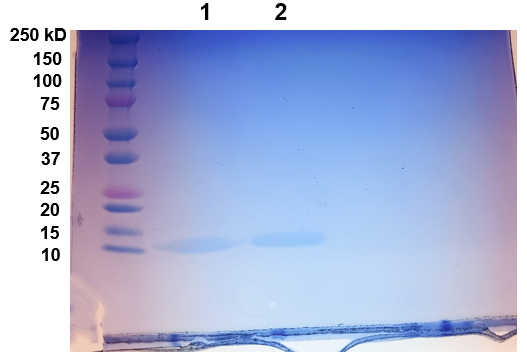


**Supplementary Fig. 12**. **Oxidative cross-linking studies of hCEACAM1 with nickel:** SDS-PAGE showing no changes in hCEACAM1 molecular weight across various lanes either in 400 µM hCEACAM1-IgV with 250 µM NiCl_2_ (lane 1) or in 400 µM hCEACAM1-IgV with 250 µM NiCl_2_ and 800 µM KHSO5 (lane 2). Molecular weight marker is shown in lane a.


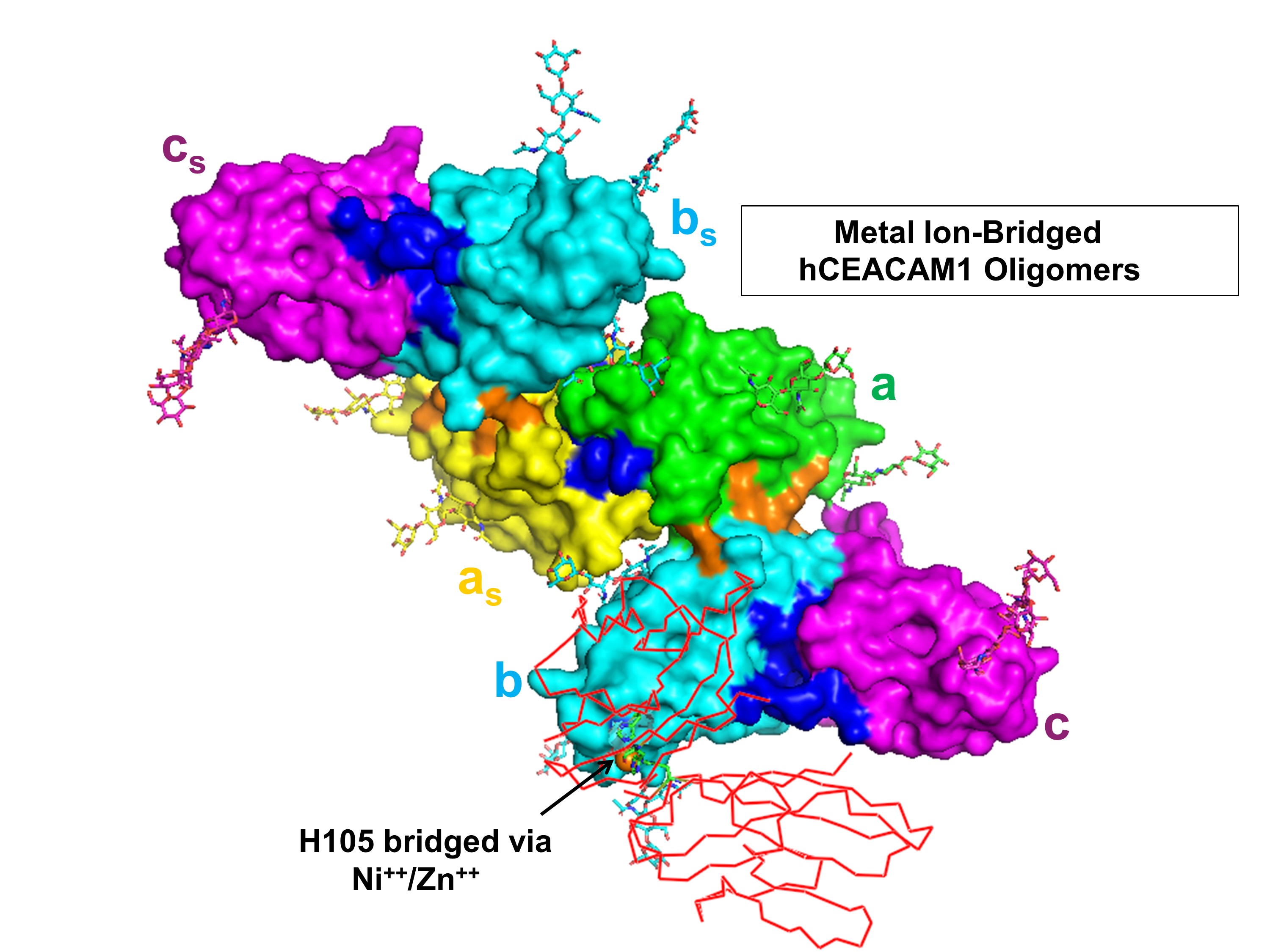


**Supplementary Fig. 13.** **Metal Ion-bridged hCEACAM1 oligomers.** Model showing formation of hCEACAM1 oligomers through primary GFCC’ face (colored as blue surface) which enables oligomerization through flexible ABED face (colored as orange surface) interactions and bridging though metal ions (Ni^++^ or Zn^++^, shown by orange sphere and coordinating hCEACA1 H105 residues in green stick representations). As observed in the crystal structure reported here, molecules a, b and c and their symmetry mates labeled a_s_ (yellow), b_s_ (cyan), c_s_ (magenta) form hCEACAM1 oligomers and are shown by surface model with modelled sugars on each molecule shown by stick representation. The hCEACAM1 molecules which are bridged through metal ions (Ni^++^ or Zn^++^ as observed in V39A mutant crystal structure) are shown by red ribbon representation and modeled on the crystal structure reported here by superimposition of a V39A mutant crystal structure (PDB code 6XNW).

| **Interactions** | **Molecule (a) residues (Interacting atom)** | **Molecule (b) residues (Interacting atom)** | **Distance (Å)** | **Comments** |
| --- | --- | --- | --- | --- |
| 1 | N70 (ND2) | E16 (OE1) | 2.2 | Interface area (Å^2^) of 475.4 was observed. Two hydrogen-bond interactions involved N70 residue. Similar N70 residue mediated hydrogen-bond interactions were observed in V39A mutant crystal structure (PDB code 6XNW) but not in N97A mutant crystal structure (PDB code 6XO1) which does not form GFCC’-mediated dimers. |
| 2 | Y68 (OH) | S72 (OG) | 2.6 |  |
| 3 | Y68 (OH) | N70 (ND2) | 2.6 |  |

**Supplementary Table 1: ABED face hydrogen bond interactions as observed in hCEACAM1 WT (PDB code 2GK2) crystal structure, molecules a/b.**

**Supplementary Table 2: GFCC’ face hydrogen bonded interactions as observed in**

**hCEACAM1 oligomer crystal structure (PDB code 7RPP), between molecules b/c.**

| **Interactions** | **Molecule (b) residues (Interacting atom)** | **Molecule (c) residues (Interacting atom)** | **Distance (Å)** | **Comments** |
| --- | --- | --- | --- | --- |
| **1** | L95 (O) | S32 (OG) | 3.0 | Interface area of 856.2 Å^2^ was observed with various hydrogen-bond interactions mediated by GFCC’ face residues and mostly consistent with WT hCEACAM1 homo dimer structure (PDB ID 4QXW, interface area of 824.6 Å^2^). Compared to WT dimer structure, fewer N97 and Y34 residues mediated hydrogen bond interactions were observed in the oligomer structure. However, a novel hydrogen-bond interaction between E37-R38 was observed in the oligomer structure. |
| **2** | E37 (O) | R38 (NH2) | 3.6 |  |
| **3** | E99 (OE1) | G41 (N) | 2.7 |  |
| **4** | L95 (O) | Q44 (NE2) | 2.8 |  |
| **5** | Q89 (OE1) | Q89 (NE2) | 3.0 |  |
| **6** | Y34 (OH) | Q89 (NE2) | 3.9 |  |
| **7** | Q44 (OE1) | N97(N) | 3.3 |  |
| **8** | S32 (OG) | N97 (ND2) | 3.5 |  |
| **9** | S32 (OG) | L95 (O) | 3.0 |  |
| **10** | R38 (NH1) | E37 (O) | 3.5 |  |
| **11** | G41 (N) | E99 (OE1) | 2.8 |  |
| **12** | Q44 (NE2) | L95 (O) | 2.8 |  |
| **13** | Q89 (NE2) | Q89 (OE1) | 3.1 |  |
| **14** | N97 (N) | Q44 (OE1) | 3.2 |  |
| **15** | N97 (ND2) | S32 (OG) | 3.8 |  |

**Supplementary Table 3: ABED face hydrogen bond interactions as observed in hCEACAM1 oligomer crystal structure (PDB code 7RPP) between molecules a/b.**

| **Interactions** | **Molecule (a) residues (Interacting atom)** | **Molecule (b) residues (Interacting atom)** | **Distance (Å)** | **Comments** |
| --- | --- | --- | --- | --- |
| 1 | I67 (O) | Q26 (NE2) | 3.0 | Interface area of 482.1 Å^2^ was observed with Q26, Q53, Q54 and Q76 residues forming a “four Q pocket” and mediating flexible interactions. Central interactions observed between I67-Q26 residues was also observed in a previously described N97A mutant crystal structure (PDB code 6XO1). No hydrophobic interactions were observed. |
| 2 | Q76 (OE1) | Q27 (NE2) | 3.1 |  |
| 3 | P59 (O) | Q53 (NE2) | 3.0 |  |
| 4 | G63 (O) | S93 (OG) | 2.8 |  |
| 5 | Q54 (NE2) | N23 (OD1) | 2.7 |  |
| 6 | I67(N) | Q26 (OE1) | 3.1 |  |

**Supplementary Table 4: Average B factors (side chains) as observed in hCEACAM1 oligomer crystal structure (PDB code 7RPP).**

| **Chain** | **Residue** | **Average B factor (side chains)** |
| --- | --- | --- |
| A | Q26 | 61.0 |
| A | Q27 | 64.0 |
| A | Q53 | 55.8 |
| A | Q54 | 48.6 |
| A | Q76 | 35.4 |
| B | Q26 | 28.3 |
| B | Q27 | 39.9 |
| B | Q53 | 41.5 |
| B | Q54 | 52.3 |
| B | Q76 | 38.9 |
| A | N70 | 27.3 |
| A | N77 | 46.6 |
| A | N81 | 54.4 |
| B | N70 | 24.0 |
| B | N77 | 38.5 |
| B | N81 | 44.0 |
| C | N70 | 27.6 |
| C | N77 | 42.8 |
| C | N81 | 65.9 |
