# Supplementary material for: Structural analysis of human CEACAM1 oligomerization
Source: Commun Biol. 2022 Sep 30;5:1042. doi: 10.1038/s42003-022-03996-4 (PMC9525683; doi:10.1038/s42003-022-03996-4)
Supplement: Supplementary file 3 — Description of Additional Supplementary Data [file 42003_2022_3996_MOESM3_ESM.docx]

**Description of Additional Supplementary Files**

**File name:** Supplementary Data 1

**Description:** The source data behind the Figure 6 plots in the paper.
